# The COVID-19 pandemic and its impact on medical teaching in obstetrics and gynecology—A nationwide expert survey among teaching coordinators at German university hospitals

**DOI:** 10.1371/journal.pone.0269562

**Published:** 2022-08-05

**Authors:** Maximilian Riedel, Niklas Amann, Florian Recker, André Hennigs, Sabine Heublein, Bastian Meyer, Anne Karge, Gabriel Eisenkolb, Jacqueline Lammert, Anna Graf, Evelyn Klein, Martin Weiss, Fabian Riedel

**Affiliations:** 1 Department of Gynecology and Obstetrics, Klinikum rechts der Isar, Technical University Munich (TU), Munich, Germany; 2 Department of Gynecology and Obstetrics, Friedrich–Alexander-University Erlangen–Nuremberg (FAU), Erlangen, Germany; 3 Department of Gynecology and Obstetrics, Bonn University Hospital, Bonn, Germany; 4 Department of Gynecology and Obstetrics, Heidelberg University Hospital, Heidelberg, Germany; 5 Department of Women’s Health, University of Tübingen, Tübingen, Germany; 6 NMI Natural and Medical Sciences Institute, University of Tübingen, Reutlingen, Germany; University of Eastern Finland: Ita-Suomen yliopisto, FINLAND

## Abstract

**Purpose:**

The COVID-19 pandemic has imposed severe challenges on medical education at German university hospitals. In this first German nationwide expert survey, we addressed the responsible university teaching coordinators in obstetrics and gynecology departments and investigated their experiences during the pandemic as well as their opinions on future developments, especially with regard to the broader implementation of e-learning in the standard curriculum.

**Methods:**

The questionnaire included 42 items and was disseminated among teaching coordinators at all 41 departments of obstetrics and gynecology at German university hospitals via an email that included a weblink to the online survey provider. Responses were collected between 19 April and 7 June 2021.

**Results:**

In total, 30 responses were collected from 41 departments across Germany and their respective teaching coordinators in obstetrics and gynecology. The general opinion of the medical teaching provided during the pandemic was positive, whereas the teaching quality in practical skills was considered inferior and not equivalent to the standard face-to-face curriculum. Lectures and seminars had to be substituted by remote-learning alternatives, while clinical clerkships were reduced in length and provided less patient contact. Students in their final year experienced only a few differences in the clinical and teaching routine. Teaching coordinators in obstetrics and gynecology stated that they intend to incorporate more e-learning into the curriculum in the future.

**Conclusion:**

The medical educators’ views presented here may help to complement the already-thoroughly investigated experiences of students under the restrictions of the COVID-19 pandemic. Medical educators in obstetrics and gynecology at German university hospitals have successfully established online and hybrid teaching alternatives to their standard face-to-face courses. Building on recent experiences, digitalization could help to improve future medical education.

## Introduction

Since the beginning of the COVID-19 pandemic in early 2020, profound challenges and changes in economics, politics, healthcare, and society have been ubiquitous. These abrupt developments have raised unique questions within medical schools worldwide on how to proceed with teaching under the restrictions of the pandemic [1, 2]. German university hospitals, medical faculties, and their individual departments–including obstetrics and gynecology (OB/GYN)–have had to find answers to these pressing questions. Due to state and federal legislation, face-to-face teaching had to be suspended as much as reasonably applicable beginning in March 2020 and was only recently and just temporarily able to be resumed in the winter 2021 semester [3].

Under regular conditions before the pandemic, the curriculum for medical students in OB/GYN within the federal regulations for medical faculties in Germany consisted of lectures, seminars, and clinical clerkships (*Blockpraktika*). Lectures for the whole semester took place at a lecture hall in a teacher-centered style and usually involved voluntary attendance, while compulsory small-group seminars (approx. 20–30 students) were designed for interactive discussions and critical thinking, and clerkships provided bedside teaching and hands-on clinical experience. A written examination and frequently a practical performance test–the OSCE (Objective Structured Clinical Examination)–concluded the OB/GYN teaching course [4]. In students’ final year before receiving their medical license (*Praktisches Jahr*), they could opt for a 16-week training course in a specialty, for example OB/GYN, with a strong integration into the clinical routine and workflow [5].

After the onset of the COVID-19 pandemic, concepts had to be established that offered a sufficient learning environment while simultaneously minimizing in-person contact between medical students, clinical personnel, and patients. E-learning approaches promptly emerged as practical solutions [6, 7]. E-learning is generally defined as a means for delivering information and knowledge to remote learners via electronic or online systems [8]. Its implementation can be either synchronous (e.g., live webinars) or asynchronous (e.g., video recording or screencasts). Digitalization in medical studies had only slowly gained the necessary attention in Germany before the COVID-19 pandemic [9]; by contrast, students’ preference for online learning over learning via analogous media has increasingly accelerated in recent years [10, 11]. Studies indicate that the disruption to medical education experienced during the pandemic may have served as a catalyst for advancing online teaching and learning both in Germany [12, 13] and worldwide [14–16].

In cooperation with the German interest group and organization of young physicians in training in OB/GYN (Young Forum of the German Society for Gynecology and Obstetrics) [17], we investigated the challenges to and adaptations of medical teaching during the COVID-19 pandemic as well as the future prospects of medical education in OB/GYN using a nationwide expert survey among teaching coordinators at German university hospitals. Special attention was given to the digital transformation of the curriculum. Within the German medical education system, university hospitals and their affiliated teaching hospitals are exclusively accredited to provide medical teaching, examinations, and licensing. Teaching coordinators at German university hospitals hold an official position within a department (e.g., OB/GYN) that is granted by the medical faculty, and these coordinators usually execute this position part-time in addition to their responsibilities as (senior) physicians. Usually, this position is executed by one person responsible at each medical department with relevant experience in medical teaching. In addition, these teaching coordinators frequently hold a postgraduate master’s degree in medical education. They are in charge of the organization and supervision of all theoretical and practical student courses, as well as, the development of the curriculum and implementation of innovative teaching concepts at their respective departments. Moreover, they are–along with the teaching coordinators from the other departments–part of the central decision-making body for the coordination of medical teaching at their institutions. Together with the medical teaching staff, they usually execute teaching during lectures, seminars, and hands-on courses, too.

We conceptualized a survey that addressed the teaching coordinators at the OB/GYN departments at German university hospitals because the implementation of the new online teaching concepts during the pandemic was within the individual departments’ and teachings coordinators’ responsibility. As described above, teaching coordinators transfer the legal requirements of the medical curriculum to the practical day-to-day teaching at their departments. Therefore, teaching coordinators will decisively shape medical education at their respective institutions in the future, and their decisions will be influenced by their experiences during the pandemic. We addressed three core questions in our survey: i) How smooth or complicated was the transition of the OB/GYN courses into mostly online teaching offerings? ii) What impact did the COVID-19 pandemic have on teaching OB/GYN in lectures/seminars, on clerkships, and on the final year? iii) Will e-learning assume a more relevant role in teaching OB/GYN in the future?

## Materials and methods

### Survey design and data acquisition

The questionnaire was created by the authors and used for the first time in this survey. It was disseminated via an invitational email to teaching coordinators at all 41 departments of OB/GYN at German public (n = 37) and private (n = 4) university hospitals. If the name of the teaching coordinator was known or accessible online, this person was directly addressed via email (n = 24); if the person responsible could not be found, the secretary’s office in the respective department or clinic was addressed with the request to redirect the email (n = 17). In the email, the intention of the study was outlined, and a weblink to the online survey hosted by the survey platform SurveyMonkey^®^ (Survey Monkey Inc., San Mateo, CA, USA) was provided.

Participation in the study was entirely voluntary. All data were completely anonymized. Consent to participate was informed and was obtained electronically before starting the questionnaire. Each study participant agreed electronically to data analysis prior to the beginning of the survey. Responses were collected between 19 April and 7 June 2021 (49 days in total). During this period of time, Germany experienced its third “COVID-19 wave”; the seven-day incidence dropped from 170 to below 50 infections per 100.000 people. Opening the survey for 49 days was intended to give the teaching coordinators enough time to participate and to observe a stable trend in the new COVID infections. Thus, all survey participants could give their answers under the same conditions as the situation of COVID-19 infections throughout Germany (and other countries worldwide) and the impact on teaching was very dynamic. A survey reminder was sent once to all departments via email after four weeks. No compensation was offered for participation. Survey participation was entirely voluntary, and answers were collected anonymously. If more than one teaching coordinator was in charge in one department, the email could also be addressed to the other coordinator(s). It was possible to regionally allocate the responses using the respondents’ regional postal code.

This study was conducted in accordance with the Declaration of Helsinki (2008 version). Since the acquired data stemmed from an anonymous and voluntary questionnaire, no further consultation or approval was required as outlined in the guidelines by the ethics committee of the Ludwigs-Maximilians-University Munich (LMU).

The questionnaire included 42 items in total, 15 of which were 5-point Likert-scale ratings. The participants were asked to indicate their agreement or disagreement with the statement for each item (1 = *strongly disagree (—)*, 2 = *disagree (-)*, 3 = *neither agree nor disagree (-/+)*, 4 = *agree (+)*, 5 = *strongly agree (++)*). Other questions were either dichotomous (n = 21), required a selection from a list of choices (n = 5), or were free text answers (n = 1).

### Statistical analysis

The data were evaluated descriptively using Excel (Microsoft^®^). Tables and figures were generated in Word (Microsoft^®^) and PowerPoint (Microsoft^®^). *P*-values were calculated using unpaired t-tests. *P*-values < 0.05 were defined as statistically significant.

## Results

### Characteristics of the study participants

**Fig 1:**
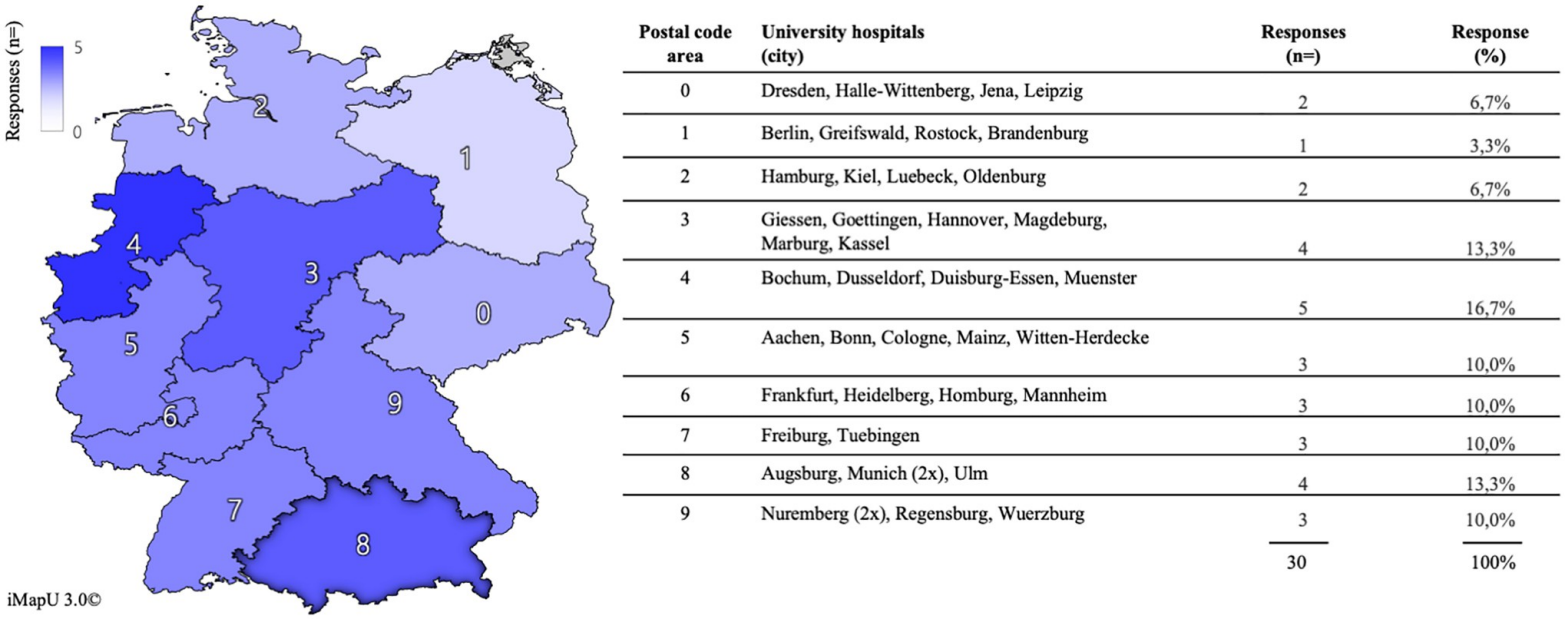
The distribution of respondents and their respective university hospitals according to their German postal codes. Graphical map of Germany in a color scheme from white (n = 0) to dark blue (n = 5) depicting the distribution of the absolute number of responses in each of the German postal code areas from 0–9 (left). Table depicting the number and locations of the German university hospitals (city), absolute responses (n =) and relative distribution (%) out of all of the 30 responses received for each postal code area (right). The map was created using iMapU 3.0 (by iEcelU) under the Creative Commons Attribution License (CCAL) CC BY 4.0 (http://creativecommons.org/licenses/by/4.0/) with permission from Carsten Tschirner.

We received n = 30 responses from a total of n = 41 departments and their respective teaching coordinators in OB/GYN. N = 27 (90%) completed the survey. According to the postal code regions, responses were distributed across all regions of Germany (Fig 1). The mean age of the teaching coordinators was 38 years (SD 7.5 years); 57% (n = 17) were male, and 43% (n = 13) were female. The majority (n = 17; 57%) had had more than 4 years of experience in their role as a teaching coordinator; 73% (n = 22) were senior physicians/consultants at their respective departments, and 23% (n = 7) were currently in specialty training.

### Assessment of the implementation of e-learning in medical teaching during the COVID-19 pandemic

The general assessment of the new online teaching offerings by the teaching coordinators was positive. Most of the survey participants (58%, n = 15) reported that they either “agreed” or “strongly agreed” with the statement that the implementation of the online modules had been swift and uncomplicated. Furthermore, they considered both the organizational and technical support by the faculty or university to be sufficient (mean Likert scale between 3.23 and 3.76). By contrast, the instructions and specifications by the faculty on how to conduct the online teaching were considered to have been less detailed and helpful (mean Likert scale 2.54). Only a minority (7%; n = 2) of the teaching coordinators “disagreed” or “strongly disagreed” with the statement that the students were satisfied with the provided online teaching. Regarding the question how equivalent the teaching quality was before and during the pandemic, a significant difference (*p*<0.001) was reported between theoretical (mean Likert scale 3.12) and practical teaching (mean Likert scale 1.84). Whereas 45% (n = 12) of the survey participants “agreed” or “strongly agreed” with the statement that the acquisition of theoretical knowledge during the pandemic was equivalent to that in normal conditions, the same assessment was reported for the practical experiences by only 4% (n = 1) of the participants. Exam results after the implementation of the e-learning modules were reported to have been comparable to those from regular face-to-face curricula (mean Likert scale 3.86) (Table 1).

**Table 1:** Evaluation of the implementation of e-learning in medical teaching during the COVID-19 pandemic.

	n =	Likert Scale (weighted average)	SD	n/a (n =)
The implementation of the e-learning offering was swift and uncomplicated.	26	3.54	1.21	0
The technical support by the university and faculty was sufficient.	26	3.42	1.1	0
The organizational support by the university and faculty was sufficient.	25	3.23	1.07	0
The technical resources and equipment for e-learning have improved in recent months.	26	3.76	1.09	0
The faculty or university provided detailed and helpful instructions for implementing e-learning.	26	2.54	0.9	0
Students were generally satisfied with the provided e-learning offerings.	26	3.68	0.94	1
The acquisition of theoretical knowledge was the same as compared to the regular curriculum.	26	3.12	1.07	0
The students’ practical experiences were the same as compared to the regular curriculum.	25	1.84	0.75	0
Recent exam results were similar as compared to the results before the pandemic.	26	3.86	0.71	4

### Impact of the COVID-19 pandemic on teaching in lectures/seminars, on clerkships, and on the final year at German university hospitals

During the COVID-19 pandemic, lectures were completely (100%) transformed into online offerings because face-to-face lectures had been suspended. Screencasts with audio narration (74%) and online webinars (78%) were frequently provided as alternative offers. The concept of “virtual patients” was mentioned as a further course alternative once. No further “experimental” teaching formats were mentioned. Only 33% of the seminars could take place in person, and a majority of the seminars (92%) were offered as online webinars (Table 2).

**Table 2:** The impact of the COVID-19 pandemic on lectures and seminars in medical teaching.

	yes		no		n/a	
	n =	%	n =	%	n =	%
Lectures could take place in person.	0	0	27	100	0	0
Lectures were replaced by screencasts with audio narration.	20	74	6	22	4	1
Lectures were replaced by online webinars.	21	78	6	22	0	0
Seminars could take place in person.	9	33	18	67	0	0
Seminars were replaced by online webinars.	24	92	2	8	0	0

A third of the participants reported that clinical clerkships had had to be entirely cancelled. If the clinical clerkships could take place, 67% of them were in person with hygiene precautions, and 23% were replaced by online webinars. Most of the participants (78%) reported that the length of the clerkship had to be reduced. During these clerkships, the teaching of practical skills (without patient contact) could take place, and patient contact was possible (both 74%). The OSCE for assessing practical skills had to be suspended in 63% of cases during the pandemic (Table 3).

**Table 3:** The impact of the COVID-19 pandemic on clinical clerkships (Blockpraktika) in medical teaching.

	yes		no		n/a	
	n =	%	n =	%	n =	%
Clinical clerkships were entirely canceled.	9	33	17	63	1	4
Clinical clerkships could take place in person (with hygiene precautions).	18	67	9	33	0	0
Clinical clerkships had to take place as online webinars.	6	23	20	77	0	0
Practical skills (without patient contact) could be taught in the clinical clerkship.	20	74	7	26	0	0
The length of the clinical clerkship was reduced.	21	78	5	19	1	4
Patient contact was possible in the clinical clerkship (with hygiene precautions).	20	74	7	26	0	0
A practical graded performance test (e.g., OSCE) was conducted.	9	33	17	63	1	4

Participants reported that final-year students were engaged in regular patient care (100%), morning meetings (89%), ward rounds (100%), outpatient consultations (96%), and operations (100%). Interdisciplinary (63%) and specialty-specific (78%) seminars could frequently take place for final year students. At the time of the survey, COVID-19 vaccinations were being offered to final-year students at most of the surveyed institutions (81%) (Table 4).

**Table 4:** The impact of the COVID-19 pandemic on the final year of medical studies.

	yes		no		n/a	
	n =	%	n =	%	n =	%
Students were engaged in regular patient care.	27	100	0	0	0	0
Students were present in regular morning meetings.	24	89	2	7	1	4
Students took part in regular ward rounds.	27	100	0	0	0	0
Students took part in regular interdisciplinary tumor boards.	23	85	3	11	1	4
Students were present in general ambulances and outpatient consultations.	26	96	1	4	0	0
Students assisted at operations.	26	100	0	0	0	0
Seminars for final-year students in OB&GYN took place (including online).	21	78	6	22	0	0
Interdisciplinary seminars for final-year students took place (including online).	17	63	6	22	4	15
All students were offered COVID-19 vaccinations.	22	81	4	15	1	4

### Future prospect of e-learning at German university hospitals

Significantly more teaching coordinators in OB/GYN believed that e-learning offerings could adequately replace lectures (mean Likert scale 3.24) as compared with seminars (mean Likert scale 2.68; *p* = 0.043) or clinical clerkships (mean Likert scale 1.69; *p*<0.001). 71% (n = 21) of participants either “agreed” or “strongly agreed” to offer more e-learning courses–also independent of the COVID-19 pandemic–in the future. Teaching coordinators in our survey were significantly more (*p*<0.001) considering transforming face-to-face lectures (mean Likert scale 3.78) than seminars (mean Likert scale 2.87) into an online or hybrid format in the future. (Table 5).

**Table 5:** Future e-learning prospects in OB/GYN at German university hospitals.

	n =	Likert Scale (weighted average)	SD	n/a (n =)
E-learning offerings could replace lectures adequately.	25	3.24	1.1	0
E-learning offerings could replace seminars adequately.	25	2.68	1.16	0
E-learning offerings could replace clerkships adequately.	26	1.69	1.09	0
We are going to offer lectures in the future–independent of COVID-19 –in an online or hybrid form.	25	3.78	0.72	2
We are going to offer seminars in the future–independent of COVID-19 –in an online or hybrid form.	25	2.87	1.01	2
We are going to offer more e-learning courses in the future for medical teaching.	26	4.04	0.59	1

## Discussion

The results of our survey provide new insights into the challenges imposed on medical education following the COVID-19 pandemic. According to the participants of this survey, the necessary adaptations to online- and remote learning had been conducted in a swift and uncomplicated manner. This finding is in line with the literature, which indicates that e-learning technologies are easy to adopt and may lead to increased faculty satisfaction [18, 19]. Moreover, the universities and medical faculties provided sufficient technical support for practically implementing the curriculum modifications. The lack of instructions or specifications regarding how to design e-learning modules may be explained by the necessary speed with which the adaptations were made in 2020/2021 as well as by the individual requirements of the different departments, which did not allow for a “one-size-fits-all” solution. By contrast, individual solutions had to be found that took into account the specific characteristics of the specialty and the traditional teaching concept of the department.

Medical faculties in Germany are used to and capable of shaping medical education independently and autonomously. The “catalogue of educational objectives in medical education” *(Gegenstandskatalog)* and the “medical licensing regulations” (*Approbation*) define the exam-relevant learning goals and set a frame with regard to the structure, quality, and quantity of medical courses (lectures, seminars, clerkships, final year, etc.). In that frame, faculties can develop and shape their teaching to a certain extent individually as long as they adhere to the federal regulations [20]. At the moment, Germany is undergoing a process of implementing the “national competency-based learning objectives for undergraduate medical education” (NKLM) which is meant to contribute to improvements in the quality of teaching and learning in medicine [21]. However, for legal certainty and to provide adequate preparation for the centralized state examination, faculties endeavor to adhere rather strictly to the regulations and provisions for the curriculum. Therefore, in general, a strong homology of the medical courses and the learning objectives is seen among medical faculties. Interestingly, a majority of the medical faculties in OB/GYN provided rather classical e-learning resources (screencasts and webinars) as opposed to more experimental digital learning methods, an example of which would be online “flipped-classroom” approaches [22]. The reason for this might again have been the speed with which the necessary adaptations were made and the lack of experience in alternative teaching formats before the pandemic.

The OB/GYN departments at German university hospitals sought to provide patient contact both in the clerkships and during the final year. Hygiene precautions and general instructions (e.g., course size) had to be followed as outlined by the regulations of the federal German university rectors’ conference [3]. A high vaccination rate against COVID-19 among hospital personnel had already been achieved in the first half of 2021 [23]. It was impractical, however, for all students to participate in regular bedside teaching without restrictions or for the experience to be able to be substituted adequately by remote learning. For final-year students, the learning experience was very similar to normal. This assessment is relevant since direct interaction between students on the one hand and patients and physicians on the other hand is essential for transferring theoretical knowledge and the therapeutic concepts into applicable clinical skills and patient care [24, 25]. By contrast, clinical clerkships were more negatively affected by the lack of both direct patient contact and hands-on training. For most medical students in Germany, these clerkships are the only opportunity to obtain real-world insights into a given specialty and to gain practical experience in this field. Studies indicate that positive experiences, for example during clerkships, are important for arousing students’ interest in and engagement with a specialty in greater depth [26, 27]. Positive experiences with medical classes also play a decisive role in final career decisions [28, 29]. As recently published, the need for online teaching methods that could also compensate for both reduced bedside teaching opportunities and less patient interaction has led to new and more experimental teaching concepts. Examples are realistic e-learning cases in a symptom-based curriculum in internal medicine at a German university hospital [30, p. 19], the use of instant messaging (e.g., via WhatsApp^®^) for distance teaching in a sub-Saharan African setting [31], or the implementation of virtual “serious gaming” as an alternative to intensive small-group teaching [32]. Exactly what impact the different quantity and quality of teaching as well as students’ satisfaction with the curriculum during the pandemic will have on career choices remains to be seen.

Whereas a majority of the teaching coordinators in our study reported a high degree of student satisfaction with the provided e-learning offerings, recent publications on the actual students’ point of view convey a more mixed picture. While some studies during the COVID-19 pandemic have reported that students prefer traditional face-to-face teaching over e-learning alternatives [33–35], other publications before the onset and during the pandemic also stressed the positive aspects and benefits of remote learning more clearly [36–39]. In contrast to the rather positive student satisfaction with the new teaching concepts reported by the respondents in our survey, in a study among 841 German medical students, 80% reported that their medical training had been negatively affected by the lack of both direct patient interaction and presence in laboratories during the pandemic. Moreover, the lack of communication between students and faculty members as well as the missing technical competencies among the educators were criticized [40]. Similar to our survey, a majority of the medical educators in the above-mentioned study noted that especially the teaching quality of practical skills had been negatively affected by the pandemic. The medical educators’ overall positive assessment of the new teaching concepts contrasts with the reported simultaneous lack of practical training experienced by the students in our survey. This discrepancy must be critically scrutinized because the quality and quantity of hands-on experiences are highly relevant for the students’ assessment of medical courses [41]. Possible explanations may be that the teaching coordinators had underestimated the importance of hands-on and practical experience for medical students’ satisfaction with their studies, or that no adequate feedback from the students to the teaching coordinators had existed. According to the literature, the teaching format–online or in-person–does not affect the acquisition of theoretical knowledge [42], though the in-person teaching format is advantageous in terms of students’ ability to form an identity or adopt a professional role as a physician [43]. Likewise, the teaching coordinators in our survey reported that the written exam results had not been negatively affected by remote learning.

Despite the problems and obstacles that have arisen from the implementation of e-learning in the last 1.5 years, a majority of the teaching coordinators in OB/GYN want to offer more e-learning courses–also independent of the COVID-19 pandemic–at their departments in the future. These teaching coordinators are aware of the general shortcomings of online teaching courses and therefore consider lectures–but not seminars or clinical clerkships–to be suitable for transforming into an online or hybrid format. In this context, two aspects are crucial. First, our own comparative analysis of medical students’ learning experiences during the COVID-19 pandemic in 2021 found that the direct interaction with the lecturer or the ability to ask questions were only relevant for a minority of our students. In addition, medical students reported little interest and low presence at face-to-face lectures before the COVID-19 pandemic [39]. Accordingly, in a survey among American osteopathic students, a large proportion of time spent during face-to-face lectures was used to study for other classes or was spent on social media or reading emails [44]. We hypothesize that the teaching coordinators in our study differentiated–similar to the medical students–between the requirements of small-group learning in interactive seminars and the passive delivery of information during lectures. Attending medical lectures is usually non-compulsory in Germany; an online or hybrid lecture-style teaching format could be a more flexible and student-oriented alternative with potentially better learning outcomes [45]. Second, the acquisition of practical skills cannot be adequately substituted by e-learning alternatives alone. It is self-evident that (practical) clinical skills require observation and repetition (under guidance) to pass through the developmental stages of novice, to competence, proficiency, and mastery. Physical presence is a necessary condition for that. However, hybrid formats have been proposed that may facilitate these steps because e-learning could serve as a time-efficient adjunct in the curriculum for teaching practical skills. For example, e-learning could help to study the theoretical basics of surgical skills [46, 47] or to establish tools for clinical problem-solving [48, 49].

The Internet has been the main driver of communication in the last two decades in all aspects of everyday life (e.g., shopping, leisure time, games, communication, etc.). As e-learning reflects this continuous advancement of digital communication, it is reasonable to hold the position that e-learning should be more integrated into the standard curriculum at German medical faculties. Online learning platforms–such as AMBOSS^®^ (AMBOSS GmbH, Berlin) [50]–have gained increasing popularity both in Germany and internationally and now constitute core learning resources for medical students. On a legal level, medical education in Germany has already been in a transitional phase for several years before the COVID-19 pandemic. The federal legislation “2020 master plan medical studies” (*Masterplan Medizinstudium 2020)* has tried to identify central future challenges in medical education and has stipulated reforms making medical studies more practical, patient-oriented, integrated, and give communication and social skills more relevance [51]. The benefits of this competency-based education are eminent [52]; however, they also require significantly more teaching resources in a time of an intensely growing medical curriculum in the last decade [53]. Likewise, the trend has accelerated internationally to reduce the amount of lecture-style teaching in a big auditorium and substitute it with more self-directed and practical learning alternatives to promote individual learning [54, 55]. Taking advantage of more digital asynchronous teaching methods (e.g., recorded lectures/screencasts) could save department resources and may thus leave more time for direct patient contact and bedside teaching during clinical courses.

From the German medical educators’ perspective, the COVID-19 pandemic is seen to be an accelerator for curricular changes with regard to the use of (online) technology in the future. The joint position paper of the “German medical faculty day” (MFT) and the “association for medical education” (GMA) has proposed a road map for the promotion of digital learning concepts. Those concepts are currently implemented and include i) the incorporation of digital learning concepts into the federal medical licensing and exam regulations, ii) the expansion and sustainable funding of digital learning resources and IT infrastructure at medical faculties, iii) the clarification of necessary legal issues with regard to data privacy and accessibility, and iiii) the didactic training of medical educators in the use of new technologies [56]. On a practical level, especially blended learning concepts have been proposed to be continued after the pandemic based upon the recent experiences [57]. The term blended learning describes a didactic teaching concept that synergistically interlinks online (e.g., videoconferencing and lecture recordings) and offline (e.g., bedside teaching or skills training) teaching formats by using it in different stages of the learning process. This promotes interprofessional and low-threshold teaching at different skill levels. Other, more sophisticated e-learning concepts, for example extended reality (XR), have not yet achieved broader recognition and acceptance in Germany [12].

It is very likely that comparable conclusions might be drawn also at the international level as most health systems have faced similar challenges during the pandemic. Similar to the broader acceptance and awareness of working from home by online applications, there will be no return to the status quo of medical teaching before the pandemic, in particular, with regard to the use of (online) technology [58]. Modern medical education will be competency-driven and rely less on lecture-style teaching. The growing implementation of entrustable professional activities (EPAs) in medical education, for example in the USA or the Netherlands, is a clear indicator for this [59, 60]. In that context, e-learning is ideally suited to transfer the necessary fundamentals in a time-efficient manner to the current generation of digitally native students before they are challenged with face-to-face contact with patients. If applied correctly, online or hybrid models can help students very efficiently to become medical professionals with a level of clinical skills and a solid foundation of theoretical knowledge.

### Limitations

The questionnaire in our survey was used for the first time and had not been previously validated. Further research approaches in the future may use additional qualitative methods (e.g., structured interviews). Our cohort of teaching coordinators formed a homogenous group as they belonged to the same specialty of OB/GYN. Further investigations could incorporate other specialties in order to draw further conclusions with regard to similarities and differences between the specialties in medical teaching during the pandemic. Another limitation of our study could lie in the time of the data acquisition (19 April to 7 June 2021). During this time, COVID-19 cases in Germany were slowly dropping during the "third wave” and their all-time maximum at around December 2020, and the participants had already been living under pandemic-related restrictions for more than a year. Thus, all teaching coordinators experienced a “steady-state” of the COVID pandemic without the influence of sudden or local events that may have an impact on decision making in the survey. The high availability of vaccines against COVID-19 has particularly allowed hybrid teaching models with a substantial amount of patient contact to be implemented. However, we consider the setting of our survey to be advantageous as new online teaching concepts in OB/GYN had already had time to be developed, implemented, and evaluated by the surveyed departments.

Although the questionnaires were answered anonymously and individually, we could not exclude a response bias by the participants because they had to assess their own actions during the pandemic, which may have enticed them to report better results than had actually been achieved. Moreover, we were interested in the expert opinions of the teaching coordinators, and these personal opinions may or may not have necessarily correlated with objective outcomes.

## Conclusion

To the best of our knowledge, this is the first nationwide survey derived from the expert perspectives of teaching coordinators–independent of their specialty–at university hospitals in Germany who are responsible for dealing with the impact of the ongoing COVID-19 pandemic on medical education. Other than the experiences of US emergency medicine clerkship director during the pandemic [61], we could not find an equivalent investigation in the international literature. Students’ experiences and opinions concerning the changes in medical teaching during the pandemic have been addressed frequently and with good intentions because students, in particular, have had to bear the main burden of social distancing and adaptations to the curriculum. The results of the medical educators presented here may help to shed light on another perspective on curriculum changes and add to the overall picture. Medical educators in OB/GYN at German university hospitals have adapted their teaching models to the challenges of the ongoing COVID-19 pandemic. They have successfully implemented web-based classes and provided hands-on experience for their students to the greatest extent possible. In building on the experiences gained during the pandemic, e-learning could play a more significant role in the standard curriculum in the future, especially as reasonable substitute for traditional face-to-face lectures. The time is therefore ripe for a digital transformation of the traditional face-to-face curriculum, and this transformation should be maintained even after the pandemic where it is found to be reasonable and beneficial. Officials from the medical faculties will have to provide and maintain adequate support for teaching coordinators during this transition. We further believe that the conclusions derived from this study should also be applicable to other countries worldwide.

## Supporting information

S1 Data (XLSX)
